# Liquid Crystals in Tribology

**DOI:** 10.3390/ijms10094102

**Published:** 2009-09-18

**Authors:** Francisco-José Carrión, Ginés Martínez-Nicolás, Patricia Iglesias, José Sanes, María-Dolores Bermúdez

**Affiliations:** Grupo de Ciencia de Materiales e Ingeniería Metalúrgica. Departamento de Ingeniería de Materiales y Fabricación. Universidad Politécnica de Cartagena. Campus de la Muralla del Mar. C/Doctor Fleming s/n. 30202-Cartagena, Spain

**Keywords:** monomer liquid crystals, lubrication, tribology

## Abstract

Two decades ago, the literature dealing with the possible applications of low molar mass liquid crystals, also called monomer liquid crystals (MLCs), only included about 50 references. Today, thousands of papers, conference reports, books or book chapters and patents refer to the study and applications of MLCs as lubricants and lubricant additives and efforts are made to develop new commercial applications. The development of more efficient lubricants is of paramount technological and economic relevance as it is estimated that half the energy consumption is dissipated as friction. MLCs have shown their ability to form ordered boundary layers with good load-carrying capacity and to lower the friction coefficients, wear rates and contact temperature of sliding surfaces, thus contributing to increase the components service life and to save energy. This review includes the use of MLCs in lubrication, and dispersions of MLCs in conventional polymers (PDMLCs). Finally, new lubricating system composed of MLC blends with surfactants, ionic liquids or nanophases are considered.

## 1. Introduction

### 1.1. Liquid Crystals

**Figure 1.**
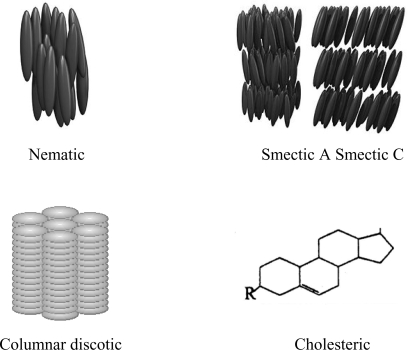
Molecular organization in some commonly used low molar mass liquid crystalline phases.

Monomer liquid crystals (MLCs) and polymer liquid crystals (PLCs) [1] are formed by mesophases which present positional and/or orientational long range order in one or two dimensions. The rigid unit responsible for the liquid crystalline behaviour is called mesogen. Figure 1 shows the molecular organization of rod-like nematic, smectic, discotic and cholesteric MLCs.

**Figure 2.**
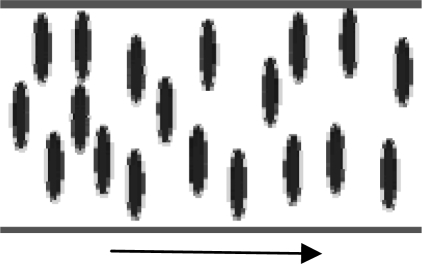
Sliding surfaces separation by MLCs.

As it is well known, thermotropic MLCs can present phase transitions due to temperature changes or the application of electric and magnetic fields. However, these mesophases can also form due to applied pressure, or sliding velocity changes when MLC molecules act as lubricant fluids separating sliding surfaces (Figure 2).

**Figure 3.**
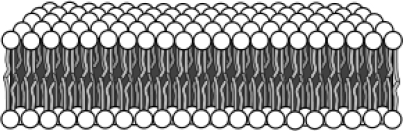
Lyotropic MLC lamellar mesophase.

Lyotropic liquid crystals form lamellar mesophases (Figure 3) within a range of concentrations in solution. Both types of MLCs have been studied as lubricants.

In this review, we will focus on the study of MLC molecules in tribology, that is, as lubricants, lubricant additives and polymer additives. The important family of wear resistant polymer liquid crystal (PLC) materials is beyond the scope of the present review.

### 1.2. Tribology

The term tribology derives from the Greek word *tribos*, meaning rubbing [2]. The objects of study of tribology are the interacting surfaces in relative motion, including friction, wear, lubrication, surface science and tribochemistry [3]. Mechanisms, machines and devices need the presence of lubricants to lower friction and contact temperatures and prevent wear, thus increasing the energy efficiency of the systems and prolonging the useful life of the components.

Lubricated tribological systems, operating under conditions of high load and low speed, frequently run in the boundary lubrication regime. Tribological systems under boundary lubrication are controlled by the tribochemical processes which take place at the interface [4].

Under these conditions the chemistry occurring within a few nanometers of the surface becomes a critical factor determining friction, wear and stick-slip. The molecular structure and composition of the lubricant, and the additives present in its formulation determine the tribological performance of the system [5]. The molecules either adsorb onto the sliding surfaces or react with the surfaces under severe conditions such as high load and low speed giving rise to protective tribolayers of low shear modulus [6,7].

Extreme pressure (EP) lubricants react with the sliding surfaces to form protective tribolayers. While surface chemistry controls antiwear performance, friction-reducing lubricants contain molecules with the ability to form stable adsorbed layers on the sliding surfaces.

## 2. Liquid Crystals in Tribology

### 2.1. Thermotropic MLCs

It has long been accepted that the presence of an adsorbed close-packed molecular monolayer can prevent direct contact between the surfaces and produce a dramatic friction reduction, while surface chemistry controls wear behaviour. The fact that liquid crystal molecules can give rise to surface-aligned ordered layers explains the interest in their use as lubricants or lubricant additives from the first studies on the effect of MLC on the lubricating properties of mineral oils [8–14].

Twenty years ago, the growing interest in the theory and applications of MLC lubricants was recognized by the celebration of the *Tribology and the liquid-crystalline state* symposium at the 198^th^ American Chemical Society Meeting. The proceedings of that symposium edited by G. Biresaw [15] include an excellent review [16] of the previous results and cover many of the main fields which have been developed since then. The studies described included the use of lyotropic and thermotropic liquid crystals presenting nematic, smectic and cholesteric mesophases (Figure 1) derived from Schiff bases, azoxys, cyanoaryls and esters. MLCs were used in pure form, as blends and as lubricant additives [17–21].

**Figure 4.**
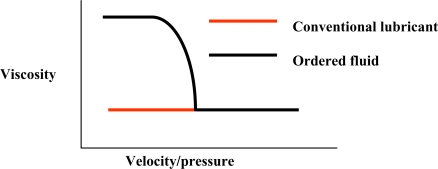
Viscosity vs velocity/pressure for conventional and ordered fluids (adapted from Ref. 22).

For conventional fluids, the thickness (h_o_) of the film separating the sliding counterpart can be estimated from the sliding velocity and the dynamic viscosity at atmospheric pressure, for each contact geometry. In contrast, in the presence of an ordered fluid such as MLC mesophases, the effective viscosity (Figure 4) of the boundary layer increases as contact pressure increases or sliding velocity decreases.

Nakano [22] studied the molecular orientation of the nematic liquid crystal 4-pentyl-4′-cyano-biphenyl (5CB) in a shear flow between parallel plates. Molecular orientation depended on the product of the sliding velocity and the film thickness. The effective viscosity was increased by low voltage for lower values of the product (sliding velocity × film thickness) with parallel orientation surfaces, which indicated the possibility of an active control of friction coefficient. With perpendicular orientation surfaces, even in the absence of applied voltage, the effective viscosity increased with the decrease of (sliding velocity × film thickness).

**Figure 5.**
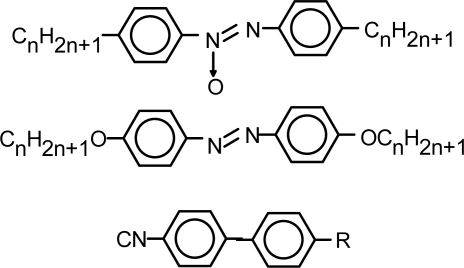
Some examples of thermotropic MLCs studies as lubricant or lubricant additives.

The formation of ordered fluid films is the base of the use of MLCs in lubrication. Some MLCs derived from azo- or cyanoaryls which were initially developed for display applications but have also been studied as lubricants or lubricant additives appear in Figure 5.

Cholesteric MLCs (Figure 1) have also received great attention, as they are present in natural biological lubricants. In fact, artificial synovial fluids containing a mixture of cholesteric liquid crystals have been described. Kupchinov *et al.* [23] studied a natural phenomenon, the low friction of living joints. The experiments revealed that the low friction of cartilages results from liquid crystalline cholesterol compounds in the synovial lubricant.

Waters, *et al.* [24] found that cholesterol palmitate liquid crystal lubricant significantly reduced wear and atomic force microscopy (AFM) showed that the liquid crystal formed protective layers on the counterface surfaces, high-nitrogen stainless-steel femoral heads and ultra-high molecular weight polyethylene (UHMWPE). It was concluded that a dramatic reduction in wear could be achieved by incorporation of liquid crystal lubricant in hip-replacement elements.

MLC molecules can reduce the friction coefficients of mobile parts and the wear rates of sliding surfaces due to their ability to orient on surfaces and form adsorbed thin films. Therefore, much attention has been paid to this aspect of MLC molecular orientation. The response of MLC molecules to electric fields was studied [22,25] and it was found that an external electric field applied across a boundary layer of nematic MLC as lubricant of steel-steel contact significantly reduced friction coefficients.

Different types of surface force apparatus (SFA) have been used [26–30] to determine the effect of confinement and shear on the positional and orientational order of thermotropic MLCs, mainly 4-cyano-4′-*n*-alkylbiphenyls confined between mica surfaces. For 4-cyano-4′-*n*-octylbiphenyl (8CB), Ruths *et al.* [26], found that the positional order increases as surface separation decreases and the orientational ordering increases with increasing shear rate under increasing sliding velocity. Artsyukhovich *et al.* [27], found a critical shear stress anisotropy for a 10.2 Å thick layer for 8CB confined between mica surfaces, with lower values for shear along the molecular axis than in the perpendicular direction.

In the case of 4-cyano-4′-*n*-hexylbiphenyl (6CB), a surface force balance was used [30], to distinguish between planar, planar twisted and homeotropic molecular orientations on mica surfaces and transitions between them as a function of water adsorption from ambient atmosphere.

The formation of self-organized monolayer MLC domains on mica was also studied by contact mode AFM and friction force microscopy [31]. The friction asymmetry of MLC monolayers was attributed to molecular tilt.

The high cost of MLCs, designed and synthesized with the high purity required for display applications has prevented until now the commercial development of MLC lubricants for mechanical machines and devices. A strategy to reduce costs, developed from early studies, has been the use of MLCs as lubricant additives in mineral and synthetic oils rather than as neat lubricant.

Kupchinov *et al.* [32] described the results of investigations performed on the lubricity of liquid crystals and their solutions in petroleum and synthetic oils as a function of MLC concentration. The MLCs present in the lubricant lead to a decrease in friction coefficient and wear.

Attempts to develop industrial applications were carried out from early studies [33], finding that the addition of cholesterol-derived fatty acid ester liquid crystals to oils reduced the running-in (the initial period of high friction) of gearboxes of metal-cutting machine tools. Lubrication of bearings was also studied [34], finding that the presence of liquid-crystalline phases could provide effective lubrication even outside the range of transition temperatures.

In order to rationalize the design of new MLC lubricants, several studies [26–30,35] have been dedicated to establish a relationship between MLCs molecular structure and tribological properties.

Cyanophenyls and cyanobiphenyls (Figure 5) are among the most widely studied MLC lubricants, due to their availability and their electrooptical applications. Using a shear force apparatus with 4-cyano-4-*n*-alkylbiphenyls (n = 5, 6, 8) it was found [29] that the films of these compounds exposed to friction depend on their structure and on the intermolecular interactions.

Mori and Iwata [35] studied the tribological behaviour of nematic and smectic liquid crystals alkyl cyanobiphenyl (CB), alkoxycyanobiphenyl (ECB) and alkylcyanophenyl cyclohexane (CPC), using a two-roller friction tester. Each liquid crystal presented a flexible structure (alkyl chain) and a rigid structure (cyanophenyl group). The frictional coefficient of the test samples was lower and the estimated film thickness was larger than those of synthetic oils. The friction coefficient was not affected by the number of carbons of the alkyl chain, but was closely dependent on the molecular structure of the rigid part of the MLC. The frictional coefficient for CB, which has a biphenyl group, was lower than that of CPC. The molecular orientation of the rigid group was observed under shearing conditions. It was concluded that the flat structure of the biphenyl group plays an important role on the tribological behaviour.

Janik *et al.* [36] used a high sensitivity surface force balance (SFB) to measure normal and shear or frictional forces for 4-cyano-4′-hexabiphenyl nematogen (6CB) confined between two mica surfaces. Results show that the highly confined nematogen (thickness in the range from 16 to ca. 100 Å) behaves under shear in a quasi-solidlike fashion for all three orientations studied: planar, planar twisted, and homeotropic.

Several families of thermotropic MLCs, 4,4′-dialkyl- and dialkoxyazobenzenes, 4,4′-diakylazoxy-benzenes and cyanoaryls (Figure 5), have been studied as commercial oil additives [37]. Tribological studies have been carried out on a real-time pin-on-disk machine at variable temperature and concentration. Variations in bulk viscosity and transition temperatures have no significant effect on friction values. Friction values for increasing concentration solutions of liquid crystals in base oil are very similar. A 1 wt% addition of liquid crystals to two different base oils lowers friction coefficients of steel for all 14 liquid crystals studied. Wear rates of steel-steel and steel-aluminium contacts are significantly lowered by addition of MLCs to the base oils.

Yao *et al.* [38] studied the use of the nematic liquid crystal 4-*n*-pentyl-4′-cyanobiphenyl (5CB) as a lubricant additive in hexadecane and attributed the good friction reduction, antiwear ability and load carrying capacity to the molecular alignment of the adsorbed liquid crystal film. The tribological benefits of 5CB as a lubricant additive can be attributed to the ordered molecular alignment of the adsorbed liquid crystal film in boundary lubrication.

Shen *et al.* [39,40] showed that when cholesterol ester MLCs are added to *n*-hexadecane, the transition between boundary to elastohydrodynamic observed for pure *n*-hexadecane when rolling speed varies from 30 to 70 mms^−1^ disappears. Film thickness was found to be related to the number of carbon atoms in the MLC alkyl chain, the polarity and concentration of the additive. Changes in film thickness with external electric field DC voltage were observed until about 30 nm.

The results indicate that the practical film thickness of hexadecane with liquid crystal is 3–5 times as large as that expected from elastohydrodynamic lubrication (EHL) theory in the low speed region. The film thickness increases with the enhancement in polarity and concentration of MLC in hexadecane, and external DC voltage. The effective viscosity of lubricant is related to film thickness and applied voltage. The film thickness increases with increasing polarity and MLC concentration. The higher ordered degree of molecules close to solid surfaces gives rise to a thicker film.

The technique of relative optical interference intensity (ROII) was used to investigate the effect of molecular order degree of cholesterol esters in hexadecane on lubricating properties in thin film lubrication (TFL) regime. When cholesterol MLC was added into hexadecane, the transition phenomenon disappears. The film thickness is closely related to the number of carbon atoms in MLC alkyl chain, the MLC polarity, the concentration of MLC in hexadecane liquid, and the applied external DC voltage. When the thickness of these lubricant films increases to about 30 nm, it nearly keeps constant and hardly changes with the voltage.

The tribochemical behaviour of the mesogenic compound (4-butylcyclohexanoic acid *p*-hexaoxy-phenyl ester) was investigated by means of a ball-on-disk machine at ambient and elevated temperatures [41]. Results show that the mesogen dissolved in *n*-hexadecane decreases the temperature of the lubricant and is an effective antiwear additive due to tribochemical processes.

Wazynska *et al.* [42] have recently described the use of mixtures of nematic and smectic liquid crystalline phases as external lubricants of steel-steel contacts. The analysis of the values of friction coefficients has shown that the friction coefficient of liquid crystalline mixtures of nematic as well as smectic A, B and E phases is lower than that of paraffin oil. Nematic mixtures have a low friction coefficient under higher loads than smectic B. Nematic and smectic MLCs have also been used [43] as additives of paraffin oil in concentrations of 0.5, 1 and 2% in the lubrication of steel. In all cases, the results show hat liquid crystals reduce the friction coefficients with respect to paraffin oil.

Of particular interest is the search for new lubricants for aluminium-steel contacts, due to the poor tribological performance of aluminium alloys and the lack of efficient lubricants for these materials. MLCs have shown their ability to reduce friction and wear of aluminium, showing in some cases, a better performance as additives than in the neat state. When the molecular polarity of the MLC additives increases, the tribological performance at high temperature and/or load is improved, due to the higher stability of the adsorbed surface layers.

Friction and wear of aluminium-steel contacts have been determined [44] using pin-on-disk tests under variable conditions of normal applied load, sliding speed and temperature, in the presence of a lubricating base oil modified with a 1 wt% proportion of three different liquid crystalline additives. The tribological behaviour of the ionic liquid crystal *n*-dodecylammonium chloride has been compared with that of two neutral liquid crystals: a non-polar species, 4,4′-dibutylazobenzene, which had previously shown its ability to lower friction and wear of metallic pairs as compared to the base oil, and a cholesterol derivative, cholesteryl linoleate. At low temperature and low sliding speed values, the friction coefficients obtained for 4,4′-dibutylazobenzene are lower than those of *n*-dodecylammonium chloride. As the severity of the contact conditions increases, this tendency reverses and the ionic species gives rise to lower friction values than the neutral one. Wear volume losses under increasing normal loads are always lower in the presence of the ionic additive.

### 2.2. Lyotropic Liquid Crystals

Lyotropic liquid crystals present a lamellar structure of amphiphilic layers (Figure 2) separated by polar solvents, such as water or alcohols. Lyotropic MLCs form organized structures in solution within a certain range of concentration.

Friberg *et al.* [19] carried out one of the first studies on lubrication with lyotropic liquid crystals. Fuller *et al.* [45] formulated new mixtures based on cetyltrimethylammonium chloride in ethylene glycol and glycerol solvents, with oleic acid, and alcohol as cosurfactants.

Currently used mineral or synthetic lubricants contain harmful or toxic components such as polycyclic aromatic hydrocarbons. Additives present in these lubricants add heavy metals and other elements such as halides or phosphorus. Environmental regulations make necessary to develop new ecological lubricants and lubricant additives. In this context, water would be an advisable lubricant base. However, its poor lubricating ability and high corrosiveness make it necessary the use of additives and modifiers.

Ma *et al.* [46] studied the use of lyotropic MLCs, in particular, the mixture diethanol ammoniumdodecanoate (DCD)/n-butanol/paraffin oil (LP)/water and found a better performance than that of a commercial lubricant.

Boschkova *et al.* [47] found that the lamellar liquid crystalline phase adsorbs on the steel surface forming a lubricating tribofilm. Poor performance is obtained when the lubricating system is in a single phase, *i.e.,* in this case a lamellar liquid crystalline region. However, good lubrication is found when the lamellar liquid crystalline phase is dispersed in water. This is attributed to a low viscosity of the system rendering a fast relaxation of the system in order to form a new film after the disturbing action of the two sliding surfaces.

The X-ray surface force technique was used [48] to monitor shear-induced orientational transitions in a lyotropic lubricant. Evidence of the formation of a boundary layer at the shearing surface was obtained. In a series of recent works, Sulek *et al.* [49–51] have studied the use of aqueous solutions containing ethoxylated esters of fatty acids or alkyl polyglucosides, recording tribological improvements even for additive concentrations as low as 0.1 wt%. The good lubricating performance of the new solutions is attributed to the ability of the additives to form liquid crystalline structures at the surface.

Amphiphilic compounds composed of a hydrophilic part and a hydrophobic alkyl chain were investigated. The compounds, sodium lauryl sulfate (SLS) and ethoxylated sodium lauryl sulfate (ESLS), exhibit strong affinity for solid surfaces and form liquid crystalline structures in water.

Very recently [52], Ma *et al.* have described the induced amphotropic and thermotropic ionic liquid crystallinity in n-alkylphosphonium halides by the addition of methanol. The results were interpreted in terms of the lengths of the three n-alkyl chains attached to the phosphorus cation, the nature of the halide anion, the influence of H-bonding interactions at the head group regions of the layered phases, and other solvent-solute interactions.

### 2.3. MLCs as Lubricants of Polymers. Polymer Dispersed MLCs (PDMLCs)

Although for most practical applications tribological uses of polymers are made in a dry environment, MLCs have also been investigated as external lubricants of polymers.

Uniform alignment of MLC molecules is a critical factor in displays technology. Different studies [53–55] have demonstrated the homogeneous alignment of MLCs on rubbed polymer surfaces, particularly on polyimide and polypirrole, and their possible applications in tribology. It has been shown [56] that the addition of MLC in low concentration increases the melt flow and the molecular mobility of the polymers, thus improving processing and ductility.

In the case of tribological properties, a possible strategy to improve the wear resistance of polymers is the addition of low molar mass or MLCs to conventional polymers to obtain polymer-dispersed liquid crystals [57–60]. In these PDMLCs, the mesophases are added in very low proportions, in contrast with the first MLC/polymer blends which contained up to a 60 wt% of MLC [56].

Dispersion of low molar mass thermotropic MLCs in polymer matrices such as polystyrene (PS), styreneacrylonitrile (SAN) [57] and polyamide (PA 6) [58] has been used as a simple way to obtain new materials with improved friction and wear resistance in sliding against steel. In the case of PA 6, MLC blends (PA 6/1 wt% MLC) show better tribological performance than the corresponding blends with a solid commercial lubricant such as MoS_2_.

Abrasive wear resistance is an important factor to improve surface finish and service life of polymers. Evaluation of abrasive wear resistance of polymers is carried out using scratch tests to determine, among other parameters, the instantaneous penetration of the indenter into the polymer as a function of applied load and sliding velocity and the residual depth (R_d_) of the abrasive groove on the polymer after the viscoelastic recovery. The addition of MLCs has been studied as a useful way to reduce surface damage due to abrasion.

Brostow *et al.* [60] described the modification of poly(ethylene terephthalate) (PET) by addition of 0.6 molar fraction of *p*-hydroxybenzoic acid (PHB). PHB is a longitudinal PLC with the mesogenic structures oriented along the main chain backbone. The best tribological results, in this case scratch resistance, were obtained for samples aligned along an externally applied magnetic field.

In the case of brittle polymers such as polystyrene [59], the presence of a thermotropic MLC additive in such a low concentration as 1 wt% induces a strain hardening effect after a number of successive scratches. This strain hardening effect under multiscratching is not present in the neat polymer. The abrasive wear resistance of PS increases as the concentration of MLC increases.

## 3. Recent Developments and Conclusion

We have seen that MLCs are advanced lubricants able to give very low friction coefficients and wear rates once they form surface aligned oriented thin films. However, some major problems which must be solved before a wide commercial implementation of these lubricants can develop. Some of these are high cost, limited conditions of effective lubrication (low temperatures, mild load and speed ranges) and low thermal stability.

New applications of MLCs in nanotribology appear attractive, as they could be effective lubricants where conventional oils can not be used. The combination of MLCs with other ordered fluids such as room-temperature ionic liquids (RTIL) [61,62], and with nanophases, such as nanoparticles [63–68] and nanotubes [69], are opening new lines of investigation. Particularly interesting are the new systems which can be effective in aqueous media.

During the last decade an increasing interest has been raised on the excellent lubricating ability of (RTILs) [61,62]. The lubricating ability of new lamellar structures has been investigated by Ge *et al.* [70] determining the effect of water and surfactant contents on the lamellar mesophase, in the presence of the RTIL 1-butyl-3-methylimidazolium hexafluorophosphate. The antiwear ability is enhanced with increasing amounts of the surfactant, while increasing water contents has detrimental effects. Increasing water content increases interlayer space in the lamellar mesophase due to the penetration of water molecules into the amphiphile bilayer.

Very recently, the lubricating ability of multiwalled carbon nanotubes (MWCNTs) evenly dispersed within hexagonal lyotropic MLC formed in the RTIL ethylammonium nitrate, has been described [69].

At the present moment, efforts are directed to the commercialization of lubricants containing MLC additives for industrial applications [71,72]. These attempts are based on reducing the costs by using less pure MLCs than those used in display applications, and could open a new era in liquid crystal tribology.
